# Female African elephant rumbles differ between populations and sympatric social groups

**DOI:** 10.1098/rsos.241264

**Published:** 2024-09-25

**Authors:** Michael A. Pardo, David S. Lolchuragi, Joyce Poole, Petter Granli, Cynthia Moss, Iain Douglas-Hamilton, George Wittemyer

**Affiliations:** ^1^ Department of Fish, Wildlife, and Conservation Biology, Colorado State University, Fort Collins, CO, USA; ^2^ Save The Elephants, Nairobi, Kenya; ^3^ ElephantVoices, Sandefjord, Norway; ^4^ Amboseli Elephant Research Project, Nairobi, Kenya

**Keywords:** elephant, vocal communication, vocal geographic variation, vocal group signature, vocal learning, vocal dialect

## Abstract

Vocalizations often vary in structure within a species, from the individual to population level. Vocal differences among social groups and populations can provide insight into biological processes such as vocal learning and evolutionary divergence, with important conservation implications. As vocal learners of conservation concern, intraspecific vocal variation is of particular interest in elephants. We recorded calls from individuals in multiple, wild elephant social groups in two distinct Kenyan populations. We used machine learning to investigate vocal differentiation among individual callers, core groups, bond groups (collections of core groups) and populations. We found clear evidence for vocal distinctiveness at the individual and population level, and evidence for much subtler vocal differences among social groups. Social group membership was a better predictor of call similarity than genetic relatedness, suggesting that subtle vocal differences among social groups may be learned. Vocal divergence among populations and social groups has conservation implications for the effects of social disruption and translocation of elephants.

## 1. Introduction

Many vocal animals exhibit intraspecific variation in the properties of their vocalizations. Geographic variation in vocalizations occurs on a wide range of spatial scales and may be graded or discrete. The songs of red-faced cisticolas (*Cisticola erythrops*) vary gradually across sub-Saharan Africa over more than 6500 km [1], while mourning warblers (*Geothlypis philadelphia*) have discrete, regionally specific dialects spanning up to 2000 km [2]. At the other end of the geographical scale, little hermit hummingbirds (*Phaethornis longuemareus*) exhibit multiple microgeographic dialects within a single lek that span just a few tens of metres [3]. Geographic vocal variation can be adaptive; for example, by facilitating mating with locally adapted individuals [4] or optimizing vocalizations for the sound propagation properties and noise profile of the local habitat [5]. However, it can also be selectively neutral [6,7].

Animals may also exhibit vocal differences among social groups that overlap in home range but are socially distinct from one another. Some cetaceans have group-specific repertoires of discrete acoustic signals, in which some but not all call types are shared with other social groups [8–10]. More commonly, vocal group signatures result from a single species-wide call type being slightly more similar in structure among members of the same group [11–17].

A common function of vocal group signatures is to facilitate social recognition. While individual recognition allows more fine-scale discrimination among conspecifics [18], group recognition may be less cognitively demanding, as it requires learning fewer distinct signals. A shared signature of group identity can act as a ‘password’, allowing animals to easily assess the group membership of others without having to recognize them all individually [14,19,20].

Geographic vocal variation can occur regardless of whether calls are innate or learned. Differences in body size or other morphological characteristics between populations can lead to geographic vocal differences, as vocal parameters such as fundamental frequency, formants and maximum duration are tied to vocal cord mass, vocal tract length and lung capacity, respectively [21]. Social learning of vocalizations often results in distinct regional dialects even in the presence of substantial gene flow [22]. Animals with some degree of behavioural plasticity in vocal production may adjust their vocalizations to propagate more efficiently in the local habitat even if they do not socially learn their calls, although learning ability seems to facilitate such acoustic adaptation [5,23,24]. Most documented examples of vocal differences among sympatric social groups involve social learning [12,13,17,25], although it is theoretically possible for vocal group signatures to be genetic.

Vocal learning can be broadly divided into usage learning and production learning, both of which can lead to vocal divergence among groups [13,26,27]. Vocal usage learning involves either learning to modify the context in which existing calls are produced or learning to modify the temporal patterning of calls, while vocal production learning involves modifying the acoustic structure of vocalizations based on auditory experience [27]. Vocal production learning is rarer than usage learning and exists on a spectrum of complexity [27]. Some animals, such as meerkats (*Suricata suricatta*) [28], domestic goats (*Capra hircus*) [16] and non-human primates [29], can learn slight modifications to species-typical vocalizations that are otherwise innate. Other vocal learners, such as swamp sparrows (*Melospiza georgiana*), require auditory input to develop normal songs but only learn songs that are typical of their species [30], while still others, such as superb lyrebirds (*Menura novaehollandiae*), can mimic heterospecific sounds [31].

**Figure 1.**
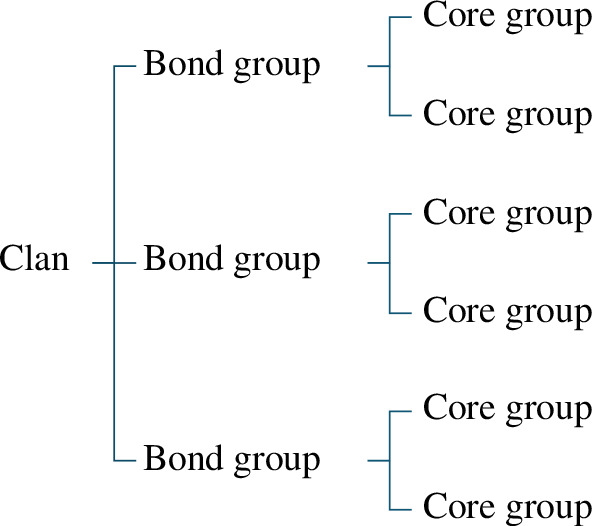
Illustration of hierarchically nested social organization of female African savannah elephants. Wittemyer *et al*. [35] reported a mean of 7.46 individuals per core group, 2.0 core groups per bond group and 3.25 bond groups per clan in Samburu.

Intraspecific vocal variation is of interest in elephants for both practical and theoretical reasons. As managers frequently translocate elephants between populations, understanding how elephant populations and social groups differ in their vocal behaviour could be valuable for their conservation [32]. Moreover, studies on captive individuals have shown that elephants are among the few mammals capable of mimicking heterospecific sounds [33,34], but it is unknown how vocal production learning manifests in wild elephants. One possibility is that elephants evolved vocal learning to facilitate social recognition through the development of group signatures, which could be particularly beneficial for elephants given their large and multi-tiered social networks (figure 1).

In African savannah elephants (*Loxodonta africana*), mothers and their dependent offspring form the most fundamental unit of social organization, multiple (usually related) mother–offspring units form a ‘core group’ led by the oldest adult female, multiple (usually related) core groups form a ‘bond group’, and multiple bond groups form a ‘clan’ [35–38]. Elephants vocally discriminate among different tiers of social affiliates, and if vocal group signatures exist this might facilitate recognition, especially of distant affiliates [39].

Animals with multi-tiered social structures may exhibit group signatures at one or more levels of social organization. In killer whales (*Orcinus orca*) and sperm whales (*Physeter macrocephalus*), different tiers of social organization can be distinguished by the number of call types they have in common, with individuals from the same core unit sharing the most call types [8,40]. In greater spear-nosed bats (*Phyllostomus hastatus*), calls cluster by social group within a cave, and all groups from the same cave cluster more closely with one another than with calls from other caves [14]. By contrast, geladas (*Theropithecus gelada*) exhibit call convergence at the level of the band (the second tier of social organization) but not at the level of smaller social units within a band [17]. Most elephant vocalizations are low-frequency, harmonic calls known as rumbles [41]. Rumbles are individually specific [42–46], but no prior study has investigated whether rumbles also exhibit group-specific acoustic signatures at any level of social affiliation or intraspecific geographic variation.

We tested the hypotheses that the acoustic structure of rumbles produced by female African savannah elephants differs between populations, bond groups, core groups and individuals. We also hypothesized that vocal differences among social groups are learned rather than genetically determined. The predictions associated with these hypotheses are summarized in table 1.

**Table 1.** Hypotheses and predictions tested in this study. The majority classifier is a model that always guesses the most numerous category in the training data, and is a more conservative baseline than the weighted expectation (chance).

hypotheses	predictions
1. Rumbles differ between allopatric populations	1a. Calls can be assigned to population with better accuracy than majority classifier
	1b. Calls from the same population are more similar on average than calls from different populations
2. Rumbles exhibit acoustic signatures at the bond group level	2a. Calls can be assigned to bond group with better accuracy than majority classifier
	2b. Calls from different core groups in the same bond group are more similar on average than calls from different bond groups
3. Rumbles exhibit acoustic signatures at the core group level, which are stronger than the acoustic signatures at the bond group level	3a. Calls can be assigned to core group with better accuracy than majority classifier
	3b. Calls from same core group are more similar on average than calls from different core groups in the same bond group or from different bond groups
4. Rumbles differ among individual callers	4a. Calls can be assigned to individual callers with better accuracy than majority classifier
5. Vocal differences among social groups are learned, not genetic	5a. Social group (core or bond group) membership is a significant predictor of call similarity but genetic relatedness is not

## 2. Methods

### 2.1. Data collection

We recorded rumbles from wild adult female elephants in Amboseli National Park, Kenya (‘Amboseli’) in 1986–1990 and 1997–2006 and in Samburu and Buffalo Springs National Reserves, Kenya (‘Samburu’) in November 2019–March 2020 and June 2021–April 2022. These two populations are 390 km apart with no current gene flow between them due to intervening urban development [47]. Both populations have been continuously monitored for decades and all individuals can be individually identified by external ear morphology [35,36]. We focused on adult females (10+ years of age) to ensure that any vocal differences between populations or social groups were not an artefact of age or sex. Our final dataset included calls from 21 adult females in Amboseli (mean ± s.d. age = 26.2 ± 12.9 years) and 81 adult females in Samburu (mean ± s.d. age = 25.6 ± 11.2 years). The field recording methods for this dataset [48] have been previously published [49].

We recorded the identity of the caller and the behavioural context of each call. The caller was identified using behavioural and contextual cues, such as an open mouth, flapping ears or being the only individual who was not a young calf in the immediate vicinity (calf calls are easily distinguished from adult/subadult calls due to their higher frequency and shorter duration) [41]. We only included in the analysis calls for which we were able to identify the caller with certainty. Behavioural context was originally scored using slightly different ethograms in Amboseli [41] and Samburu [49]. To facilitate comparison between these two datasets, we concatenated behavioural context into nine categories shared across both populations (electronic supplementary material, table S1).

### 2.2. Definition of social groups

We determined the group membership of the elephants in Samburu following a previously published protocol [35]. In brief, we calculated simple ratio association indices [50] between all adult females in the population using observational data collected between January 2019 and April 2022, excluding individuals that were seen less than 20 times during this period. We performed a Ward’s hierarchical cluster analysis on association indices, plotted the cumulative number of bifurcations as a function of bifurcation distance, and identified the most significant knot by visual inspection of the plot (*sensu* [35]). We cut the dendrogram at the height corresponding to this knot and designated all clusters below this height as separate core groups. To identify bond groups, we repeated the procedure using only the matriarch (oldest female) of each core group. We did not include Amboseli data in the analysis of core groups and bond groups because we did not have comparable association data for Amboseli, and because 92% of our Amboseli recordings came from individuals belonging to the same (subjectively defined) core group. Data analysis was conducted using R v. 4.2.2 [51].

### 2.3. Call measurement

We measured 94 acoustic features on each call describing the distribution of energy across time and frequency in the mel spectrogram (electronic supplementary material, table S2, Supplemental Methods). A mel spectrogram is similar to a traditional spectrogram (raster plot with time on the *x*-axis, frequency on the *y*-axis, and amplitude indicated by pixel darkness) but with frequency transformed to the logarithmic mel scale [52]. While the mel scale was designed to approximate human hearing sensitivity, most other mammals, including elephants, perceive frequency on a similar logarithmic scale [53].

While the measurements we took from the mel spectrogram capture more of the variation in the calls than traditional measurements such as fundamental frequency and formants, they also capture background noise and thus may be more susceptible to influence from the recording equipment. To ensure that differences between Amboseli and Samburu could not be attributed to the different recording gear used in each population, we also traced the second harmonic (*f_1_*) contour of each call, which is highly robust to recording equipment differences [54]. We calculated nine summary statistics of the *f_1_* contour (electronic supplementary material, table S2).

### 2.4. Individual, social unit and population assignment accuracy

To test the hypothesis that Amboseli and Samburu elephants exhibit population-level acoustic differences, we ran a random forest (500 trees, six variables/node, 60% of observations/tree, minimum node size = 1, no maximum tree depth) to predict population as a function of the mel spectrogram acoustic features. A random forest is a machine learning model comprising many decision trees, each using a randomly selected fraction of the data [55]. This analysis included 938 calls from 81 individuals in Samburu, and 414 calls from 21 individuals in Amboseli. As random forests are biased towards more numerous classes [56], we balanced the dataset by randomly subsampling the Samburu observations so there were an equal number of observations in each class. To ensure that the model could only predict population using cues that generalized across callers, we randomly selected 20% of the callers with at least five calls each from each population and allocated all calls from these callers to the test set, with the remaining calls allocated to the training set [57]. We calculated the proportion of observations in the test set that were classified correctly (classification accuracy) and ran a one-tailed exact binomial test comparing the classification accuracy with the proportion that would have been classified accurately if the model always guessed the most common group in the training set (‘majority’ or ‘zero rate’ classifier). The majority classifier is a standard baseline model used in machine learning [58]. When there are the same number of observations in each class, the majority classifier is mathematically equivalent to the weighted expectation (guessing each class with a probability equal to the proportion of the data comprised by that class). However, when the data are imbalanced, the majority classifier always outperforms the weighted expectation. We repeated the above process 10 000 times and calculated the median *p*-value across all runs. The number of calls allocated to the test set varied across runs because different callers produced different numbers of calls. The mean ± s.d. proportion of the calls allocated to the test set across 10 000 runs was 0.16 ± 0.05. Note that although the full dataset was balanced by subsampling an equal number of calls from each population, the data were not perfectly balanced within the training and test sets, because the number of calls from each population allocated to the test set depended on the number of calls per caller. Moreover, the majority population in the training set was not always the majority population in the test set. Thus, the majority classifier accuracy could be greater or less than 50%.

To determine if vocal differences between the two populations could be an artefact of the different recording equipment used in each population, we ran the same model using the *f_1_* contour measurements instead of the mel spectrogram measurements. We also ran a logistic regression model with population as the response variable and the *f_1_* contour measurements and caller age as the regressors, to determine which acoustic features had a significant relationship to population.

To test the hypotheses that elephants exhibit vocal signatures of group identity at the bond group or core group level, we ran two additional random forest models (same hyperparameters) to predict bond group and core group, respectively, as a function of the mel spectrogram acoustic features, using only data from Samburu. To ensure that the bond group model could only use acoustic features that generalized across the entire bond group, rather than features specific to core groups, to predict bond group, we restricted the dataset to bond groups that contained at least two core groups with at least five calls each in our dataset (six bond groups, 931 calls) [57]. For each iteration of the model, we randomly selected one core group from each bond group and allocated all calls from those core groups to the test set. To ensure that the core group model could only use acoustic features that generalized across the entire core group, rather than features specific to individual callers, to predict core group, we restricted the dataset to core groups that contained at least two individuals with at least five calls each in our dataset (seven core groups, 794 calls) [57]. For each iteration of the model, we randomly selected 20% of the callers with at least five calls each from each core group and allocated all calls from those individuals to the test set. The data were severely imbalanced across bond groups and core groups, but we were unable to balance it by subsampling the more numerous classes because doing so would have reduced the sample size excessively. We ran 10 000 iterations for each model, calculating the classification accuracy and *p*-value for each run as before. The mean ± s.d. proportion of the calls allocated to the test set was 0.32 ± 0.17 for the bond group model and 0.23 ± 0.08 for the core group model.

To test the hypothesis that elephant rumbles are individually specific, we ran a fifth random forest (same hyperparameters) to predict individual caller identity as a function of the mel spectrogram acoustic features. As calls produced by the same caller on the same date might exhibit similar features due to temporary circumstances such as the caller’s internal state, behavioural context and ambient conditions, we randomly selected one date for each caller and held out all calls from these caller dates as the test set [49,57]. We used callers from both populations for this analysis, but only included callers that produced at least three calls on at least two different dates each (427 calls from 15 callers in Samburu, 218 calls from nine callers in Amboseli). We calculated the classification accuracy and *p-*value for each of 10 000 iterations as before. The mean ± s.d. proportion of calls allocated to the test set was 0.20 ± 0.02.

### 2.5. Assessment of call similarity

Elephant rumbles vary with the behavioural context and the individual identity and age of the caller [41,59,60]. To determine if there were acoustic differences among populations, bond groups or core groups that could not be explained by behavioural context, caller identity or caller age, we calculated random forest proximity scores between each possible pair of calls. The random forest proximity score for a given pair of calls was the proportion of trees for which both calls were classified in the same terminal node, adjusted for the size of the node, and represented a metric of call similarity in terms of the acoustic features most relevant to predicting the response variable [61]. We calculated proximity scores from four different random forests (population with mel spectrogram features, population with *f_1_* contour features, bond group and core group), using the same hyperparameters and subsets of the data as before except that we increased the number of trees to 8000 and did not balance the data by subsampling or hold out any observations as a test set (population models: all observations, *n* = 1352; bond group model: all bond groups in Samburu containing at least two core groups with at least five calls each, *n* = 931; core group model: all core groups in Samburu containing at least two individuals with at least five calls each, *n* = 794).

For each set of proximity scores, we ran a generalized linear mixed model with a gamma error distribution and a log link function. In each model, pairwise call proximity score was the response variable and pair ID (unique identifier for a given pair of callers) was a random effect. Fixed effects were ‘pair class’ (whether the two calls in a given pair came from the same population, bond group or core group, depending on the model) and the scaled and centred absolute value of the age difference between the two callers. For the population models, the ‘pair class’ variable was binary: a pair of calls could either be from the same population or not. For the bond group and core group models, the ‘pair class’ variable had three possible states: same core group, different core groups within the same bond group or different bond groups within the Samburu population. To ensure that differences between populations or social groups were not an artefact of individual identity or behavioural context, we only included pairs of calls with the same behavioural context and different callers. We also only included calls for which we were certain of the behavioural context (see electronic supplementary material, Supplemental Methods). This resulted in a sample size of 149 640 call pairs for the two population models, 97 586 call pairs for the bond group model and 72 437 call pairs for the core group model. As proximity scores could be 0, we added 0.00001 to all proximity scores so all the values would be positive. For the bond group and core group models, we used the R package ‘emmeans’ [62] to examine the pairwise contrasts for each level of the ‘pair class’ variable.

To assess whether vocal similarity between individuals was better explained by social affiliation or genetic relatedness, we ran two additional gamma regressions on call pairs, modelling call proximity score as a function of ‘binary pair class’ (see below), caller age difference and caller genetic relatedness. One gamma model used proximity scores extracted from the random forest trained to predict core groups and the other used proximity scores extracted from the random forest trained to predict bond groups. These two sets of proximity scores represented the pairwise similarity between calls in terms of the features most relevant to predicting core group membership or bond group membership, respectively. For the model using proximity scores extracted from the bond group random forest, binary pair class indicated whether the two calls in a pair were from the same bond group. For the model using proximity scores extracted from the core group random forest, binary pair class indicated whether the two calls in a pair were from the same core group. These gamma models could only be run on the subset of callers for which we had genetic relatedness data (13 individuals for the bond group model, eight for the core group model). Genetic relatedness was calculated in a previously published study using 20 microsatellite loci extracted from tissue or dung samples collected before 2006 [37]. Due to social disruption caused by poaching, many core groups and bond groups in the Samburu population now include unrelated individuals, which made it possible to independently assess the effects of social affiliation and relatedness on call similarity [37].

All statistical analyses were performed in R v. 4.2.2 [51] and 0.05 was used as the significance threshold for all tests.

## 3. Results

### 3.1. Vocal differences between allopatric populations

**Figure 2.**
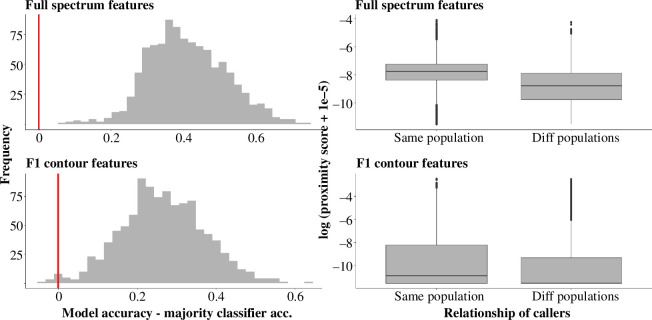
Evidence for acoustic differences between Amboseli and Samburu populations. Top row: models using acoustic features derived from mel spectrogram. Bottom row: models using acoustic features derived from second harmonic contour. Histograms represent distribution of classification accuracies of 10 000 random forest models trained to predict population from the acoustic features. Positive values (to the right of the red line) indicate that the random forest outperformed the majority classifier (binomial exact tests; median *p* < 0.0001 for both models). Boxplots represent acoustic similarity of calls within versus between populations (gamma regression; *p* < 0.0001 for both models). Acoustic similarity is represented by call proximity scores extracted from the random forest. The log of the proximity scores is plotted on the *y*-axis to facilitate visualization of the data (less negative values = greater call similarity). Centre lines = medians, box edges = interquartile ranges, whiskers = 1.5 × interquartile range.

**Figure 3.**
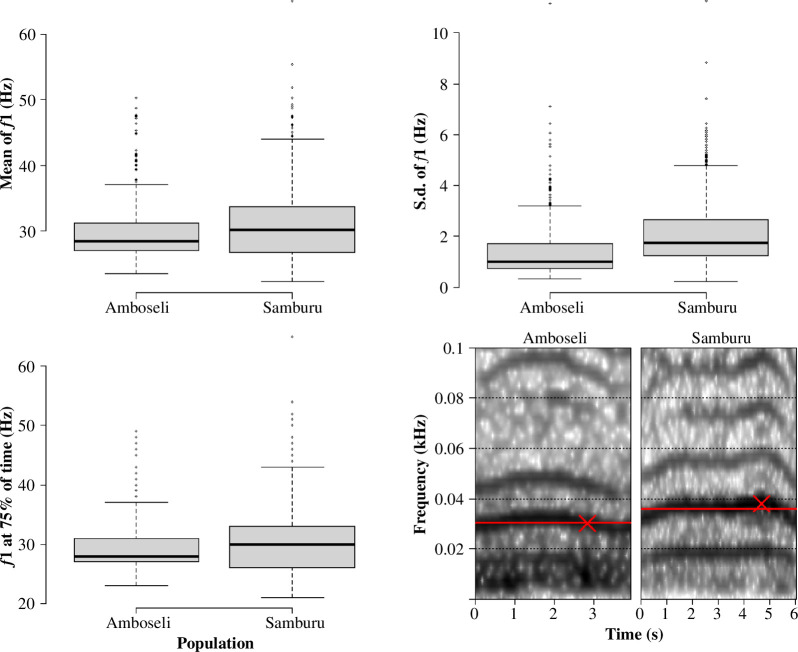
Population differences in the second harmonic (*f_1_*) contour. Boxplots represent population differences in the three features of the second harmonic that differed between Amboseli and Samburu (logistic regression; mean of second harmonic: *p* = 0.026; s.d. of second harmonic: *p* = 0.004; frequency of second harmonic at 75% of call duration: *p* < 0.0001). Bottom right: spectrograms of rumbles from the same behavioural context (contact calling) made by a 17-year-old female in each population (2 kHz sampling rate, Hanning window, 800 samples/window, 90% overlap). Red lines: mean frequency of second harmonic; red Xs: frequency of second harmonic at 75% of call duration.

The random forest model trained to predict population from mel spectrogram acoustic features was significantly more accurate than the majority classifier that always guessed the population with the most calls in the training set (median observed classification accuracy = 0.79, median majority classifier accuracy = 0.39, median *p* < 0.0001) (figure 2). Similarly, the random forest model trained to predict population from *f_1_* contour features, which describe less of the variation in the call but are robust to influences of the recording equipment, was significantly more accurate than the majority classifier (median observed classification accuracy = 0.65, median majority classifier accuracy = 0.39, median *p* < 0.0001) (figure 2). Three parameters of the *f_1_* contour had a significant relationship with population in a logistic regression model after controlling for caller age: mean of the second harmonic (*χ* ^2^ = 5.0, *p* = 0.026), s.d. of the second harmonic (*χ* ^2^ = 8.3, *p* = 0.004), and value of the second harmonic at 75% of call duration (*χ* ^2^ = 16.9, *p* < 0.0001). All three values were higher in Samburu than in Amboseli on average (figure 3, table 2).

**Table 2.** Results of logistic regression modelling population as a function of acoustic features derived from the second harmonic contour (Hz) and caller age (days). *χ* ^2^ statistics and *p*-values are derived from analysis of deviance on the model. Significant *p*-values in bold.

regressor	coefficient	*χ* ^2^ statistic	*p*‐value
mean of second harmonic	−0.664	4.96	**0.026**
s.d. of second harmonic	1.16	8.31	**0.004**
skew of second harmonic	−0.0335	0.0628	0.802
kurtosis of second harmonic	0.0440	0.688	0.407
10th percentile of second harmonic	−0.000548	0.0000	0.997
90th percentile of second harmonic	0.0101	0.0027	0.959
frequency at 25% of call duration	0.0617	0.513	0.474
frequency at 50% of call duration	0.0758	0.675	0.411
frequency at 75% of call duration	0.358	16.9	**<0.0001**
caller age	−0.0000692	19.6	**<0.0001**

Calls from different individuals within the same population were significantly more similar than calls from different populations after controlling for behavioural context, individual caller identity and the age difference between the callers (gamma regression; proximity scores calculated from mel spectrogram features: *χ* ^2^ = 493.0, *p* < 0.0001; proximity scores calculated from *f_1_* contour features: *χ* ^2^ = 41.3, *p *< 0.0001) (figure 2, table 3). This indicates that the vocal divergence between Samburu and Amboseli was not merely an artefact of differences in the age structure or prevalence of certain behavioural contexts in the two populations, but rather reflects a population-specific vocal difference. Call similarity also decreased as the age difference between the callers increased (gamma regression; proximity scores calculated from mel spectrogram features: *χ* ^2^ = 35.2, *p* < 0.0001; proximity scores calculated from *f_1_* contour features: *χ* ^2^ = 80.8, *p* < 0.0001) (table 3).

**Table 3.** Results of gamma regressions with call proximity score as the response variable. Rows are separate models and columns are regressors. Values in each cell include the coefficient, *χ* ^2^ statistic, and *p*-value for fixed effects (*χ* ^2^ and *p* based on analysis of deviance) and the s.d. for random effects. Caller age was scaled to make it more comparable in range to other regressors. Pair ID represented a unique pair of callers.

model #	RF used to generate proximity scores	levels of pair class (reference level bold)	pair class	caller age (scaled)	genetic relatedness	pair ID (random effect)
1	population ~ mel spectral features	same versus **different** population	Coef = 0.518; *χ* ^2^ = 493.0; *p* < 0.0001	Coef = − 0.059; *χ* ^2^ = 35.2; *p* < 0.0001	NA	0.535
2	population ~ F1 contour features	same versus **different** population	Coef = 0.381; *χ* ^2^ = 41.3; *p *< 0.0001	Coef = −0.233; *χ* ^2^ = 80.8; *p *< 0.0001	NA	5.074
3	bond group ~ mel spectral features	same core group versus same bond group versus **different bond groups**	Coef (same core group) = 0.181; Coef (same bond group) = 0.049; *χ* ^2^ = 13.2; *p* = 0.001	Coef = −0.111; *χ* ^2^ = 68.0; *p *< 0.0001	NA	0.658
4	core group ~ mel spectral features	same core group versus same bond group versus **different bond groups**	Coef (same core group) = 0.228; Coef (same bond group) = −0.061; *χ* ^2^ = 26.5; *p < *0.0001	Coef = −0.154; χ^2^ = 82.8; *p <* 0.0001	NA	0.543
5	bond group ~ mel spectral features	same versus **different** bond group	Coef = 0.309; *χ* ^2^ = 3.54; *p* = 0.060	Coef = −0.048; *χ* ^2^ = 2.13; *p* = 0.144	Coef = 0.146; *χ* ^2^ = 0.133; *p* = 0.715	0.508
6	core group ~ mel spectral features	same versus **different** core group	Coef = 0.374; *χ* ^2^ = 5.85; *p* = 0.016	Coef = 0.013; *χ* ^2^ = 0.181; *p* = 0.670	Coef = 0.382; *χ* ^2^ = 1.49; *p* = 0.222	0.375

### 3.2. Vocal differences among sympatric social groups

**Figure 4.**
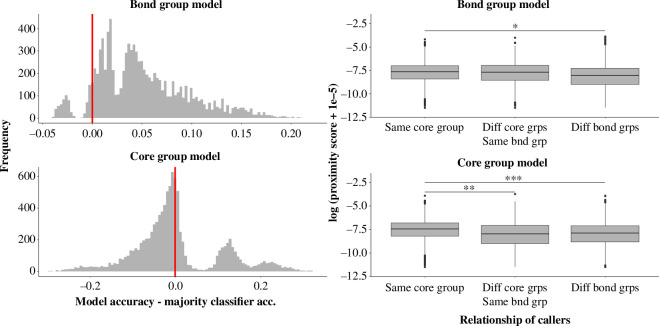
Limited evidence for vocal differences among sympatric social groups. Histograms represent distribution of classification accuracies of 10 000 random forest models trained to predict bond group or core group from the acoustic features. Positive values (to the right of the red line) indicate that the random forest outperformed the majority classifier (binomial exact tests; bond group model: median *p* = 0.001; core group model: median *p* = 0.70). Boxplots represent acoustic similarity of calls in same core group versus same bond group versus different bond groups (gamma regression, post hoc contrasts: *p < 0.01, **p < 0.001, ***p < 0.0001). Acoustic similarity is represented by call proximity scores extracted from the random forest. The log of the proximity scores is plotted on the *y*-axis to facilitate visualization of the data (less negative values = greater call similarity). Centre lines = medians, box edges = interquartile ranges, whiskers = 1.5 ∗ interquartile range.

The random forest trained to predict bond group in Samburu from mel spectral features correctly predicted bond group for 10% of calls on average, which was significantly more accurate than the majority classifier (median observed classification accuracy = 0.10, median majority classifier accuracy = 0.05, median *p* = 0.001) (figure 4). The random forest trained to predict core group in Samburu from mel spectral features correctly predicted core group for 19% of calls on average, which was no better than the majority classifier (median observed classification accuracy = 0.19, median majority classifier accuracy = 0.19, median *p* = 0.70) (figure 4). Analysis of call proximity scores indicated that calls recorded from the same core group were more similar than calls recorded from different core groups in the same bond group or calls recorded from different bond groups, suggesting some vocal convergence at the core group level (tables 3 and 4). By contrast, calls from different core groups in the same bond group were no more similar than calls from different bond groups, suggesting a lack of vocal convergence at the bond group level (tables 3 and 4). In both the bond group model and the core group model, call similarity decreased as caller age difference increased (table 3).

**Table 4.** Post hoc contrasts indicating the statistical significance of acoustic differences between core groups and bond groups. Both models in this table were of the form call proximity score (similarity score between a pair of calls) ~ pair class + scaled caller age difference + (1|pair ID), where pair class was a three-way categorical variable indicating whether the two calls in question were recorded from the same core group, different core groups in the same bond group or different bond groups. The models differed in which random forest (RF) model was used to generate the call proximity scores. In model 3, the proximity scores represented the pairwise similarity of calls in terms of the acoustic features most relevant to predicting bond group membership, while in model 4 the proximity scores represented the pairwise similarity of calls in terms of the acoustic features most relevant to predicting core group membership.

model #	RF used to generate proximity scores	same core group versus different core groups in same bond group	same core group versus different bond groups	different core groups in same bond group versus different bond groups
3	bond group ~ mel spectral features	*p* = 0.144	*p* = 0.001	*p* = 0.627
4	core group ~ mel spectral features	*p* = 0.0001	*p < *0.0001	*p* = 0.536

### 3.3. Vocal differences among individual callers

The random forest trained to predict individual caller ID from mel spectral features performed significantly better than the majority classifier (median classification accuracy = 0.29, median majority classifier accuracy = 0.03, median *p* < 0.0001) (table 3, electronic supplementary material, figure S1).

### 3.4. Social versus genetic influences on call similarity

Social group membership was a better predictor of call similarity than genetic relatedness. For the proximity scores extracted from the random forest trained to predict core group, belonging to the same core group was a significant predictor of call proximity (gamma regression, *χ* ^2^ = 5.9, *p* = 0.016), but genetic relatedness was not (gamma regression, χ^2^ = 1.5, *p* = 0.222) (electronic supplementary material, figure S2, table 3). For the proximity scores extracted from the random forest trained to predict bond group, belonging to the same bond group was a marginally non-significant predictor of call similarity (gamma regression, *χ* ^2^ = 3.5, *p* = 0.060) and genetic relatedness was not significant (gamma regression, *χ* ^2^ = 0.133, *p* = 0.715) (electronic supplementary material, figure S2, table 3).

## 4. Discussion

The vocal flexibility of elephants is notable, but few studies have assessed geographic and social group variation in wild elephant calls. Our results provide evidence that elephant rumbles differ in structure between allopatric populations and suggest at least some vocal divergence among sympatric core groups. Our results also indicate that to the extent that elephants exhibit vocal differences among social groups, these differences are probably due to social factors rather than genetically determined, as group membership, but not genetic relatedness, was a significant predictor of call similarity.

As 92% of the calls we recorded in Amboseli came from a single social unit, the effects of population and social group were somewhat confounded. However, random forest models were able to achieve much higher discriminability between the Amboseli and Samburu populations than among social groups within Samburu. This suggests that the two populations are more vocally divergent than social groups within a single population. Previous work has shown that elephant populations differ in the most common order of call combinations [63], and our present findings add to this by showing that populations also differ in the fine acoustic structure of rumbles.

There are several possible explanations for the difference in call structure between Amboseli and Samburu. Cultural evolution is a common driver of vocal differences between animal populations. For example, white-throated sparrows (*Zonotrichia albicollis*) and yellow-naped amazon parrots (*Amazona auropalliata*) exhibit regional dialects that probably stem partially from copying errors during vocal production learning [7,64]. Chimpanzees exhibit population differences in the order of call combinations that probably result from usage learning [26]. In elephants, vocal production learning seems more likely than usage learning to explain the population differences we observed, given that they were structural differences in a single call type. However, we cannot definitively rule out the possibility that Amboseli and Samburu elephants share the same repertoire of rumble subtypes but differ in the contexts in which each subtype is used, thus leading to an average difference in the fundamental frequency of rumbles recorded from the two populations.

Amboseli and Samburu exhibit significant genetic divergence in mitochondrial DNA (*F*_ST_ = 0.423, *p* < 0.0001) and to a lesser extent nuclear microsatellites (*F*_ST_ = 0.02, *p* < 0.05), so genetics may play a role as well [65]. Moreover, if Samburu elephants were more stressed than Amboseli elephants on average during the periods in which we recorded them, this might explain why Samburu rumbles were higher in pitch, as stress is correlated with elevated fundamental frequency in elephant rumbles [66]. Acoustic adaptation also cannot be definitively ruled out. If Samburu has more low-frequency noise for some reason, the Samburu elephants might have shifted the frequency of their rumbles upwards to compensate, as has been documented in many other species [67]. However, there is no obvious reason to expect differences in the acoustic environments of Samburu and Amboseli given that both are protected areas with similar habitat types surrounded by relatively low-density pastoralist communities [47].

One explanation that probably can be excluded is body size. There is little difference in growth asymptotes among female elephants in these two populations [68], and the vocal differences persisted when controlling for age. To the extent that there is any difference in body size between the populations, Samburu elephants are slightly taller on average [68], which if anything would be expected to result in lower fundamental frequencies [21]. However, we observed the opposite.

Elephants are often translocated between populations to facilitate gene flow, reduce ecological damage caused by local overcrowding or mitigate human–elephant conflict [32,69]. Our finding that elephant populations less than 400 km apart exhibit significant differences in the structure of their rumble vocalizations could thus have important implications for elephant conservation, especially if the differences are found to result from cultural or genetic divergence, rather than acute responses to local environmental conditions. A prior study found that elephants reacted to seismically transmitted playback of alarm rumbles recorded in their own population but ignored alarm rumbles from a different population, although it is unclear if this was due to failure to recognize alarm calls from another population, increased responsiveness to familiar callers or differences between the stimuli unrelated to population of origin [70]. Further study is warranted to determine whether vocal differences between elephant populations impede communication or social integration. If so, translocation efforts should attempt to quantify and account for behavioural compatibility between populations when deciding where to move individuals, to the extent that it is feasible to do so.

We found evidence for limited vocal differences among sympatric social groups. Analysis of call proximity scores indicated statistically significant vocal convergence among members of the same core group but found no evidence for vocal convergence among group members at the bond group level. Our random forest models correctly predicted the core group for 19% of calls and correctly predicted the bond group for 10% of calls, but only the bond group model was statistically significantly better than the majority classifier. This is probably because the training data were more unbalanced for the core group model on average, so the majority classifier was more accurate for this model, thus raising the threshold for a statistically significant improvement over the majority classifier.

Overall, our results suggest that elephants exhibit greater vocal convergence at the core group level than at the bond group level. This is similar to some cetaceans and bats, in which the greatest vocal convergence takes place at the closest tier of social affiliation [8,14,40]. However, it contrasts with geladas, in which vocal convergence takes place at the band level, a higher tier of social organization [17]. In geladas, bands typically forage and sleep together, and the function of call convergence at the band level is hypothesized to be maintaining social cohesion among less closely knit social affiliates who must coordinate their behaviour on a daily basis [17]. By contrast, in elephants, bond group members from different core groups are more often separate than together, so there is less opportunity and potentially less need to develop vocal convergence at the bond group level [35].

The low accuracy of the random forest models in predicting social group membership from the acoustic structure of rumbles suggests that while detectable vocal differences among social groups do exist (at least at the core group level), they probably have limited potential for facilitating social recognition. Vocal convergence among core group members in elephants may not have an adaptive function; for example, it could be a selectively neutral by-product of vocal learning that evolved for some other purpose. Meerkats have group signatures in their close calls but fail to discriminate them, indicating that vocal differences among social groups can develop even if they do not facilitate recognition [28]. Previous work has shown that elephants can discriminate between the calls of close social affiliates (core/bond group members), distant social affiliates (clan members) and non-affiliates [39]. If elephants do not rely on group signatures to make this discrimination, that means they can individually recognize the calls of at least 100 other elephants on average, including individuals with whom they have limited interaction, suggesting that they possess exceptional social memory [39].

The vocal differences we observed among elephant social groups and populations reflected minor variations in the fine structure of a single shared call type. This is more similar to the subtle group signatures found in some bats [13,71], ungulates [15,16], meerkats [28] and non-human primates [72] than to the dialects of some cetaceans and birds, where individuals from different social groups or geographical areas use categorically distinct repertoires of call/song types [2,7,8,40]. While we did not include call types other than rumbles in this study, rumbles comprise the vast majority of elephant vocalizations [41], and we have not noticed any differences among social groups or populations in the total repertoire of basic call types.

It is sometimes difficult to determine if subtle modifications to species-specific call types involve vocal production learning. For example, one study found that translocated captive chimpanzees (*Pan troglodytes*) slightly modified their food calls to be more like those of their new group members [73], but this may simply reflect changes in arousal [74]. We think it is unlikely that the vocal differences we observed among sympatric elephant core groups can be explained by group differences in arousal or other physiological states, as these groups are all subject to similar environmental stressors and we controlled for behavioural context in our analysis of call proximity scores, but we cannot conclusively rule it out.

Learning slight modifications to existing call types seems to require less neurological specialization than learning entirely new call types, which explains why many species traditionally considered non-vocal production learners, such as non-human primates, are capable of acquiring vocal group signatures [75]. Yet unlike these species, some captive elephants have displayed a remarkable ability to mimic novel heterospecific sounds, far beyond what is necessary to develop the subtle vocal convergence we observed in this study [33,34]. Similar to humans, who exhibit both subtle vocal convergence among social affiliates and more sophisticated forms of vocal production learning [72], it may be that wild elephants use vocal learning in multiple ways. The recent discovery that elephants address one another with name-like calls suggests another possible function for vocal production learning in elephants [49].

Our study replicates previous findings that rumbles are individually distinct [42–46] and that rumble structure is correlated with caller age [59]. However, our random forest model predicting individual caller identity from call structure only achieved 29% classification accuracy on average, in contrast to previous studies on captive elephants that achieved 55–60% accuracy using discriminant function analysis [42,45] and 82.5% accuracy using hidden Markov models [43]. This difference in performance may be an artefact of the number of individual callers included in each dataset (24 for our study versus 6 or 13 for previous studies). It is also possible that rumbles produced under natural conditions are more variable and therefore more difficult to assign to individual callers than rumbles produced in captivity, or that our field recordings were noisier and more difficult to classify than recordings made in captivity.

This study is among the first to investigate possible consequences of vocal learning in wild elephants and adds to a growing body of literature on vocal variation and plasticity in mammals [75,76]. Interspecific variation in elephant calls is well-documented [63,77,78], and one study described intraspecific variation in the syntax of elephant call combinations [63]. Geographic variation in vocal structure has also been documented for elephants’ closest living relatives: sirenians and hyraxes [79,80]. However, prior to the present study, intraspecific variation in the structure of a single call type was largely unexplored in elephants. Longitudinal studies on translocated individuals and individuals who change groups within a population due to poaching or other social disruption could help clarify the role of vocal learning in wild elephant communication. Playback experiments could determine if elephant calls differ in function as well as form across populations.

## Data Availability

Data and code are available from the Dryad digital repository [48]. Supplementary material is available online [81].
